# Impact of type of child growth intervention program on caregivers’ child feeding knowledge and practices: a comparative study in Ga West Municipality, Ghana

**DOI:** 10.1002/fsn3.318

**Published:** 2015-12-02

**Authors:** Faith Agbozo, Esi Colecraft, Basma Ellahi

**Affiliations:** ^1^ Department of Family and community HealthSchool of Public HealthUniversity of Health and Allied SciencesHo (Hohoe campus)Ghana; ^2^ Department of Nutrition and Food ScienceUniversity of GhanaP. O. Box LG 134LegonGhana; ^3^ Faculty of Health and Social CareUniversity of ChesterChesterCH1 4BJUnited Kingdom

**Keywords:** Caregiving, community‐based growth promotion, growth monitoring and promotion, infant and young child feeding

## Abstract

Community‐based growth promotion (CBGP) delivered by community volunteers aims at enhancing the traditional growth monitoring and promotion (GMP) program delivered by community health nurses through the promotion of optimum infant and young child feeding (IYCF) leading to improved child growth. This study compared IYCF knowledge and practices among caregiver–child pairs (0–24 months) receiving child welfare services from CBGP (*n* = 124) and GMP (*n* = 108) programs. Semistructured questionnaires were used to interview caregivers on IYCF knowledge/practices and validated food frequency questionnaire used to record infants’ food intakes. Group differences were determined using Chi‐square and independent samples *t*‐tests (*P* < 0.05; 95% confidence interval [CI]). Mean IYCF knowledge scores were similar (CBGP: 10.84 ± 1.69 vs. GMP: 10.23 ± 1.38, *P* = 0.062). However, more CBGP caregivers (17%) were highly knowledgeable than their GMP counterparts (5%) (*P* = 0.011). Early breastfeeding initiation (CBGP: 54% vs. GMP: 28%, *P* < 0.0001), exclusive breastfeeding (CBGP: 73% vs. GMP: 56%, *P* = 0.001), and timely complementary feeding (CBGP: 72% vs. GMP: 49%, *P* = 0.014) were reportedly higher among CBGP caregivers. Underweight was 11% (CBGP: 8% vs. GMP: 14%, *P* = 0.154). Mean dietary diversity scores (10 food groups) were similar (CBGP: 4.49 ± 1.89 vs. GMP: 3.87 ± 1.89, *P* = 0.057) but more CBGP caregivers (77%) achieved minimum dietary diversity than their GMP counterparts (61%) (*P* = 0.035). Few caregivers achieved minimum meal frequency (CBGP: 31% vs. GMP: 29%, *P* = 0.486) and minimum acceptable diet (CBGP: 23% vs. GMP: 21%, *P* = 0.464) indicators. Number of children under 5 years owned by caregiver (adjusted odds ratio [AOR]: 0.405; 95% CI: 1.13–78.53, *P* = 0.038), her educational level (AOR: 0.112; 95% CI: 0.02–0.90, *P* = 0.040), and IYCF knowledge (AOR: 0.140; 95% CI: 0.03–0.79, *P* = 0.026) significantly predicted optimum child feeding. Nutrition education on optimum complementary feeding and birth spacing strategies should intensify.

## Introduction

Childhood malnutrition is a serious public health problem associated with adverse health and socioeconomic consequences. Globally, an estimated 26% of children below 5 years of age were stunted, 16% were underweight and 8% were wasted in 2011 (UNICEF‐WHO‐The World Bank, 2012). The vast majority of undernourished children are in south‐central Asia and sub‐Saharan Africa. In Ghana, 28% of children under‐five years are stunted, 14% are underweight, and 9% are wasted (Ghana Statistical Service, 2008). Child growth monitoring and promotion (GMP) programs have long been identified and recommended as an effective approach to preventing child malnutrition (UNICEF, 2006).

The GMP program was introduced by UNICEF and implemented in Ghana in the 1970s with the main objective of reducing the prevalence of malnutrition (UNICEF, 1990). The earlier growth monitoring program instituted by the WHO in the 1950s was limited to weighing and plotting children's weights to detect abnormal growth (WHO, 1962). Inclusion of a promotion component in the GMP program, intended to foster communication between health workers and caregivers concerning child health and nutrition, was expected to enhance caregivers’ child caring and feeding behaviors leading to improved child growth (Pearson 1995).

However, a systematic review of studies on the program revealed that the “promotion” component of “GMP” which encompassed child‐centered caregiver counseling was poorly implemented (Pearson 1995). Also, poor attendance and low coverage were reported in UNICEF supported programs in China, Ecuador, Indonesia, Malawi, Thailand, Zaire, and Zambia (Ashworth et al. 2008). In Burkina Faso, Niger and Mozambique, fewer than 30% of mothers were counseled (Hampshire et al. 2004). Reasons cited included lack of health personnel to provide the service, inaccessible child health services and poor attendance by caregivers. However, in Ghana and other developing countries, it is has been shown that adequate caregiver counseling and longer effective participation in child growth programs is associated with improved knowledge of caregivers on infant feeding and positive anthropometric outcomes of children (Owusu and Lartey 1992; Colecraft et al. 2004; Alderman 2007).

Thus, the community‐based growth promotion (CBGP) concept was an attempt to address some of the shortcomings of the GMP program and improve coverage, delivery of services and effective participation by caregivers. The program was pioneered by the Manoff Group and promoted by UNICEF during the 1980s as a child survival strategy to engender the caregiver–health worker interaction believed to be crucial for program success (UNICEF, 1990; Mason et al. 2006). Prior to its implementation, the CBGP concept was successfully piloted in Indonesia in 1977 where caloric intake of participating children improved by 200–300 calories per day and consumption of green leafy vegetables doubled with subsequent reduction in malnutrition from 25% to 14% (Griffiths and Nobbe 1985; Griffiths et al. 1996). Following the piloting success, the CBGP concept was adopted and implemented in several countries with outcomes that suggested that the program had positive effects on knowledge, attitudes, and practices of participating families which led to improved growth of children (Van Roekel et al. 2002; Hossain et al. 2005; Galasso and Umapthi 2007; Muyeti‐Stevens 2007).

Modalities for rolling out the CBGP program in Ghana have been documented by the Ghana Health Service (Ghana Health Service, 2010). In Ghana, major differences between the CBGP and the GMP programs centers on “where,” “who,” and “how” the services are delivered. Typically, the GMP program is delivered at child welfare clinics (CWC) situated at health centers or outreach health posts and the services are provided by trained health professionals, mostly community health nurses who are sometimes supported by nutrition extension workers and auxiliary health aides. In contrast, the CBGP program is delivered at the community‐level in selected rural and deprived urban communities where the GMP program is readily unavailable and is intended to be owned by the community. Here, services are provided by community volunteers called child growth promoters who have been trained by the Ghana Health Service to carry out growth monitoring of infants and counseling of caregivers on infant and young child feeding (IYCF). The volunteers are supervised by community health nurses who carry out specialized activities such as immunizations. Another difference between the two program types is that whereas the GMP program targets children under 5 years, the CBGP program focuses on children from birth to 24 months. In both programs, monthly attendance by caregiver–child pairs is required where infant growth monitoring is carried out and caregivers are counseled on optimum IYCF practices. However, in the GMP program, the counseling is typically group‐based and prescriptive. Conversely, the counseling delivered in the CBGP program is usually individualized and participatory and the community volunteer is expected to negotiate with the caregiver and motivate her to adopt optimum IYCF practices.

After over a decade of implementation, there is little documented information on whether the CBGP program is achieving the intended impact and if it is an improvement over the traditional GMP program. This study evaluated differences in the effects of a CBGP and a GMP program on caregivers’ IYCF knowledge and practices in two rural communities in the Ga West municipality, Greater Accra region, Ghana.

## Materials and Methods

The study was a cross‐sectional survey conducted at Nsakina and Dom Sampaman, both rural communities in the Ga West municipality of the Greater Accra region, Ghana. The study targeted all caregiver–child pairs enrolled in the CBGP and GMP programs in Nsakina and Dom Sampaman communities, respectively. All caregivers who received child welfare services in either the CBGP (*N* = 124) or GMP (*N* = 108) programs in the two study locations between January and March 2012 were invited to participate and interviewed after completing an informed consent document.

### Socio‐demographic, economic, and health characteristics of caregiver–child pairs

A semistructured questionnaire was used to collect data on background characteristics of caregivers. This included questions on socio‐demographic characteristics (e.g., age, marital status, educational level, employment, household possessions, etc.) and some maternal experiences such as use of antenatal services during pregnancy, education on IYCF at antenatal and CWCs, home visits by program implementers). Additionally, data on the child's age, age at program enrollment, birth weight, and current body weight were abstracted from the child health record booklets.

### Knowledge of caregivers on IYCF

Questions intended to assess caregivers’ IYCF knowledge were designed based on IYCF counseling topics documented in the Ghana Health Service revised child health record booklet (Ghana Health Service, 2010) which ought to be communicated to caregivers during child welfare sessions. Caregivers responded to 13 knowledge questions on age‐specific IYCF practices regarding breastfeeding and complementary feeding.

### IYCF practices

A sample of the food frequency questionnaire used during the 2008 Ghana demographic and health survey was used to collect a 3‐day information on habitual dietary intakes of the children on complementary feeding (Ghana Statistical Service, 2008). The food groupings in the 3‐day questionnaire were as follows: light cereal porridges; stiff cereal‐based meals; roots/tubers/plantain; fruits/vegetables rich in vitamin A; other fruits/vegetables; animal products; milk/dairy products; legumes/nuts/oil seeds; fats/oils; and commercial baby products. The WHO core indicators that were used to assess IYCF practices were early initiation of breastfeeding; exclusive breastfeeding under 6 months; introduction of solid, semisolid or soft foods (timely initiation of complementary foods); minimum dietary diversity; minimum meal frequency, and minimum acceptable diet (WHO and UNICEF, 2010).

### Child anthropometry

Birth weight categorizations and nutritional status of the children was determined using the birth weight and current body weight measurements recorded in the child health record booklets.

### Data analyses

Data were analyzed using SPSS (version 20.0; Incorporated, Chicago, IL). Differences between the CBGP and GMP programs were determined using Chi‐squared test for categorical variables (e.g., sex, proportions of underweight children) and independent samples *t*‐test for continuous variables (e.g., age, difference in children's mean weight‐for‐age *Z*‐scores [WAZ]) at *P* < 0.05 (95% confidence interval [CI]). Infant body weight measurements were converted to WAZ using WHO Anthro software (version 3.2.2) (World Health Organization, Geneva, Switzerland). Underweight was defined as WAZ‐score <−2. Similarly, their birth weights were classified as normal (≥2.5 kg) and low (<2.5 kg). Univariate binary logistic regression model was developed for both known and unknown caregiver–child pair determining factors for child feeding according to WHO minimum IYCF standards and significant independent association (unadjusted odds ratios, UOR) determined. Variables were selected into the final model by considering independent variables with a significance of ≤0.500. In the final model, a multivariate binary logistic regression analysis was done to identify the effects of interaction of the explanatory variables (adjusted odds ratio, AOR) on the outcome variable (optimum child feeding).

### Measurement of socioeconomic status (SES) index

Variables used in the determination of socioeconomic status (SES) index were education, occupation, income, source of water supply, toilet facilities, fuel for cooking, and household possessions. Each variable was scored based on a method described by Caro and Cortes (Caro and Cortés 2012). The total possible score ranged from 26 to 88 (Table 1). The predetermined SES constituent indicators were analyzed using principal component analysis (factor analysis). The Kaiser‐Meyer‐Olkin measure of sampling adequacy statistic and Bartlett's test of sphericity tests were applied to determine the usefulness of the data for SES index rating. Based on the possible minimum and maximum values, SES index was determined by providing arbitrary cut‐offs for the cumulative scores. Total score >65 indicated high SES; 40–65, average SES and <40, low SES.

**Table 1:** Indicators and associated scores used to measure SES

SES indicator	Variable	Score
Education^a^	None or primary	1
	Junior high school	2
	Technical/vocational or senior high school	3
Occupation^a^	Housewife	22
	Trading or artisan	31
	Farming	37
Household possessions^a^	House	7
	Land	5
	Vehicle	4
	Milling machine, livestock, or motor	3 each
	Bicycle, refrigerator, sewing machine, television or farm produce	2 each
	Radio, fan or mobile phone	1 each
Income (Ghana cedis) spent on food per week^b^	<20^c^	1
	20–50	2
	>50	3
Sources of water supply^b^	Borehole or well	2
	Rainwater/river	1
Fuel for cooking^b^	Gas	3
	Charcoal	2
	Firewood	1
Household access to toilet facilities^b^	Private toilet	2
	Public latrine	1
	Open defecation	0

a These indicators were described by Caro and Cortés (2012).

b These were extra indicators added by the authors.

c Exchange rate at data collection was 1 dollar to 1.8 Ghana cedis.

### Evaluation of caregivers IYCF knowledge

Every knowledge‐based question was given a score of either zero or one for questions that demanded a “Yes” or “No” response (e.g., should colostrum be fed to newborns?) and on a 0–2 scale for questions that required caregivers to give specific examples (e.g., give examples of foods rich in vitamin A). Cumulative scores, ranging from 0 to 15 points were computed for each caregiver. Using the Ghana Health Service counseling topics as a guide, an interquartile range was determined for the knowledge scores obtained. Relying on distribution of the scores, knowledge rating cut‐offs were assigned as follows: low knowledge (0–8); fair knowledge (9–12); and high knowledge (13–15).

### Assessment of complementary feeding

The WHO core indicators used to assess complementary feeding are introduction of solid, semisolid or soft foods (timely complementary feeding); minimum dietary diversity; minimum meal frequency and minimum acceptable diet (optimum feeding) (WHO and UNICEF, 2010). The minimum dietary diversity indicator was calculated based on the proportion of children 6–24 months who received foods from four or more food groups during the previous day. The minimum meal frequency indicator was calculated as a proportion of breastfed infants 6–8 months who received at least two meals and 9–24 months who received at least three meals the previous day. The infant or young child met the minimum acceptable diet indicator if the minimum dietary diversity and minimum meal frequency indicators were both met. Based on this, the feeding was classified as either optimum or suboptimum.

### Ethical considerations

Ethical clearance was obtained from the Institutional Review Board of the Noguchi Memorial Institute for Medical Research, University of Ghana, Legon (040/11‐12). Permission was granted from the Ga West Municipal Health Management Team before commencement of study. Written informed consent was obtained from the caregivers with signature (if literate) or thumbprint (if illiterate).

## Results

### Socio‐demographic, economic, and health characteristics of caregiver–child pairs enrolled in the CBGP and GMP programs

Children in the two programs were similar in age and the average age was about 8 months (Table 2). Based on age at program enrollment, children in the GMP and CBGP programs were enrolled at average ages of 6 and 8 weeks, respectively (*P* = 0.04). Mean birth weights of children were similar. Compared to caregivers in the GMP program, the CBGP caregivers were older and more likely to have completed higher than primary level education, be married or cohabiting and had higher parity. While borehole was the main source of water for nearly 90% of the GMP caregivers’ households, over 30% of the CBGP caregivers said their households relied on wells or rainfall as their main water supply (*P* < 0001). The CBGP caregivers were significantly more likely than the GMP caregivers to use charcoal and gas rather than firewood as their main fuel source. There were no significant group differences in the other socio‐demographic variables assessed.

**Table 2:** Socio‐demographic characteristics of caregivers–child pairs

Characteristics	CBGP (*n* = 124)	GMP (*n* = 108)	*P*‐value^a^
Children's age (months)	8.3 ± 5.5	7.5 ± 4.2	0.207
Sex			
Males	61 (49.2)	52 (48.1)	0.489
Females	63 (50.8)	56 (51.9)	
Child's age at program enrollment (weeks)	8.3 ± 0.81	6.3 ± 0.63	0.042
Child's birth weight (kg)	3.08 ± 0.55	2.97 ± 0.51	0.286
Caregivers’ age (years)	27.9 ± 5.8	23.9 ± 5.1	<0.0001
Educational level			
None	19 (15.3)	21 (19.4)	0.023
Primary	25 (20.2)	34 (31.5)	
Junior high school	46 (37.1)	41 (38.0)	
Technical/vocational school	27 (21.8)	9 (8.3)	
Senior high school	7 (5.6)	3 (2.8)	
Marital status			
Married	77 (62.1)	51 (47.2)	0.007
Cohabiting	36 (29.0)	38 (35.2)	
Single	11 (8.9)	19 (17.6)	
Parity	2.8 ± 1.6	2.1 ± 1.4	0.001
Number of children under 5 years	1.6 ± 0.7	1.6 ± 0.9	0.741
Household size	4.8 ± 1.7	4.9 ± 2.0	0.860
Occupation			
Trading	69 (55.7)	45 (41.7)	0.173
Vocational^b^	29 (23.4)	30 (27.8)	
Housewife	23 (18.5)	28 (25.9)	
Farming	3 (2.4)	5 (4.6)	
Water supply			
Borehole	82 (66.2)	93 (86.1)	<0.0001
Well	37 (29.8)	6 (5.6)	
Rainwater/river	5 (4.0)	9 (8.3)	
Toilet facilities			
Private toilet	104 (83.8)	77 (71.5)	0.113
Public latrine	15 (12.2)	23 (21.3)	
Open defecation	5 (4.0)	8 (7.2)	
Cooking fuel			
Charcoal	103 (83.1)	72 (66.7)	<0.0001
Firewood	7 (5.6)	30 (27.7)	
Gas	14 (11.3)	6 (5.6)	
Weekly expenditure on food (GH¢)			
<20	6 (4.8)	13 (12.1)	0.080
20–50	23 (18.5)	24 (22.2)	
>50	95 (76.6)	71 (65.7)	
SES index			
Low	66 (53.2)	63 (58.3)	0.337
Average	58 (46.8)	45 (41.7)	

a Independent sample *t*‐test or Chi‐square test, significance (*P* < 0.05).

b Hairdressing and dressmaking.

#### Healthcare services received by caregivers

Prior to enrolling in the child growth programs, about 91% of caregivers in the CBGP program received antenatal care services during their last pregnancy compared to 71% of GMP caregivers (*P* < 0.0001). In both programs, about 90% of caregivers had received education on IYCF during their visits to antenatal and or CWCs. Compared to caregivers enrolled in the CBGP program (31%), more GMP caregivers (57%) reported having received home visits from the community health nurses (*P* < 0.001).

### IYCF knowledge of caregivers

Generally, there were no significant group differences in caregivers’ knowledge on breastfeeding initiation, frequency of breastfeeding in 24 h, duration of exclusive breastfeeding, timely complementary feeding initiation, duration of complementary feeding, minimum meal frequency and minimum dietary diversity for infants and young children (Table 3). In both groups, with the exception of timely breastfeeding initiation where approximately 50% caregivers (CBGP: 53.2% vs. GMP: 41.7%, *P* = 0.099) were aware of it being within an hour after delivery, in most cases, more than 60% of caregivers in both programs gave correct responses to the other IYCF knowledge questions asked. However, CBGP caregivers were significantly more likely to correctly mention foods rich in vitamin A and tended to have a higher mean knowledge score (CBGP: 10.84 ± 1.69 vs. GMP: 10.23 ± 1.38, *P* = 0.062). Also, significantly more CBGP caregivers were rated as having high IYCF knowledge (CBGP: 17% vs. GMP: 5%, *P* = 0.011).

**Table 3:** Infant and young child feeding knowledge of study caregivers in the CBGP and GMP programs

Knowledge assessment	CBGP (*n* = 124)	GMP (*n* = 108)	*P‐*value^a^
Breastfeeding initiation			
Within an hour after birth	66 (53.2)	45 (41.7)	0.099
Breastfeeding frequency in 24 h			
On demand	106 (85.5)	82 (75.9)	0.113
Duration of exclusive breastfeeding			
Six months	92 (74.2)	65 (60.2)	0.074
Timing of complementary feeding			
Six months	92 (74.2)	66 (61.1)	0.097
Duration of complementary feeding			
6–24 months	79 (63.7)	65 (60.2)	0.270
Minimum meal frequency per day			
6–8 months (≥2 times daily)	108 (87.1)	87 (80.6)	0.175
9–11 months (≥3 times daily)	93 (75.0)	82 (75.9)	0.870
12–24 months (≥4 times daily)	30 (24.2)	36 (33.3)	0.124
Minimum dietary diversity per day			
6–8 months (≥3 food groups)	101 (89.4)	70 (90.9)	0.730
9–11 months (≥3 food groups)	112 (90.3)	95 (88.0)	0.563
12–24 months (≥4 food groups)	114 (91.9)	93 (86.1)	0.112
Knowledge of vitamin A‐rich foods			
>2 foods mentioned	17 (13.7)	2 (1.9)	0.004
1–2 foods mentioned	24 (19.4)	20 (17.9)	
None mentioned	83 (66.9)	86 (80.2)	
Knowledge of iron‐rich foods			
>2 foods mentioned	11 (8.9)	18 (16.7)	0.099
1–2 foods mentioned	66 (53.2)	45 (41.7)	
None mentioned	47 (37.9)	45 (41.6)	
Knowledge score^b^	10.84 ± 1.69	10.23 ± 1.38	0.062
Knowledge rating			
Low	7 (5.6)	9 (8.3)	0.011
Fair	96 (77.4)	94 (87.0)	
High	21 (16.9)	5 (4.6)	

a Independent sample *t*‐test or Chi‐square test, significance (*P* < 0.05).

b The maximum score is 15.

### IYCF practices of caregivers

#### Breastfeeding practices of caregivers

With the exception of feeding colostrum to newborns, where there was no group difference in caregivers breastfeeding practices, significantly more caregivers in the CBGP program followed recommended IYCF practices (Table 4). The CBGP caregivers were significantly more likely to report initiating breastfeeding within one hour of birth (54% vs. 28%; *P* < 0.0001), practicing exclusive breastfeeding for 6 months (73% vs. 56%; *P* = 0.001) and timely initiation of complementary feeding (72% vs. 49%; *P* = 0.014). Fermented corn dough porridge was the most common first complementary food given to infants in both groups but use of commercial products for complementary feeding tended (*P* = 0.078) to be higher among the GMP caregivers.

**Table 4:** Breastfeeding practices of caregivers in the CBGP and GMP programs

Breastfeeding practices	CBGP (*n* = 124)	GMP (*n* = 108)	*P‐*value^a^
Breastfeeding initiation			
<1 h	67 (54.0)	30 (27.8)	<0.0001
1–24 h	26 (21.0)	36 (33.3)	
>24 h	31 (25.0)	42 (38.9)	
Feeding colostrum to newborns	103 (83.1)	79 (74.5)	0.112
Exclusive breastfeeding for 6 months	91 (73.4)	60 (55.6)	0.001
Pre‐lacteal feeds given to newborns			
Coconut juice	8 (6.5)	20 (18.4)	0.022
Formula	12 (9.6)	4 (3.7)	
Fluids given <6 months infants			
Water	11 (8.9)	30 (27.8)	0.002
Gripe water	19 (15.3)	14 (13.0)	
Water and gripe water	3 (2.4)	4 (3.8)	
Timely complementary feeding	57 (72.2)	32 (48.5)	0.014
First complementary food served			
Fermented corn dough porridge	55 (69.6)	33 (50.8)	0.078
Commercial products^b^	23 (29.1)	28 (43.1)	
Sugar solution	1 (1.3)	4 (6.2)	

a Chi‐square test, significance (*P* < 0.05).

b Include formula milk and cereal blends.

#### Types and frequency of consumption of complementary foods by study children aged 6–24 months

**Figure 1:**
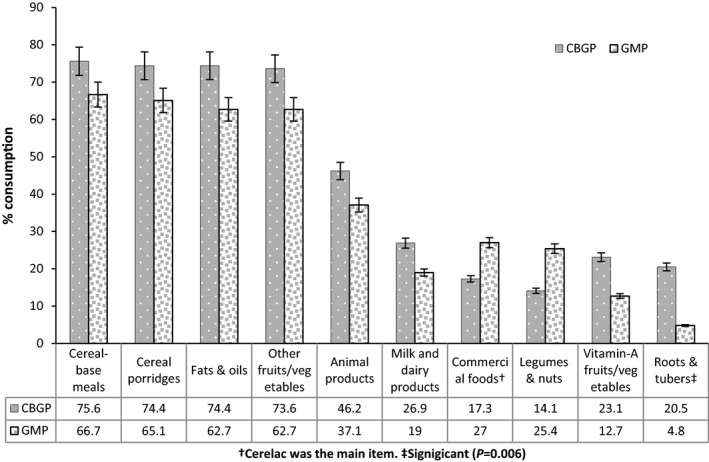
Types of complementary foods consumed by study children aged 6–24 months enrolled in the CBGP and GMP programs.

Figure 1 shows the types of foods consumed by the children age 6–24 months over the 3‐day assessment period. The most commonly consumed food groups (at least 50% consumption rate) among children in both groups were cereal‐based meals, cereal porridges, fats and oils, and nonvitamin A‐rich fruits and vegetables. Conversely, the least consumed foods (less that 30% consumption rate) were milk and dairy products, vitamin A‐rich fruits and vegetables, roots and tubers, commercial foods and legumes, and nuts. The mean dietary diversity scores of children in the CBGP program tended to be higher than those in the GMP program (CBGP: 4.49 ± 1.89 vs. GMP: 3.87 ± 1.89; *P* = 0.057).

#### Children aged 6–24 months who were fed according to the minimum IYCF guidelines

Of the 232 study children, 141 participants were aged 6–24 months and were on complementary feeding. However, only 31 (22.0%) were optimally fed. Overall, higher proportions of CBGP children were fed according to minimum IYCF guidelines (Table 5). Minimum dietary diversity (CBGP: 77% vs. GMP: 61%; *P* = 0.035) was the only IYCF indicator that over half of the caregivers were able to meet. In both programs, about a third of the caregivers met the minimum meal frequency indicator (CBGP: 31% vs. GMP: 29%; *P* = 0.486) with only a quarter meeting the minimum acceptable diet indicator to achieve optimum child feeding. (CBGP: 23% vs. GMP: 21%; *P* = 0.464).

**Table 5:** Study children between 6 and 24 months who were fed according to minimum IYCF guidelines

Minimum IYCF indicator	CBGP (*n* = 78)	GMP (*n* = 63)	*P*‐value^a^
Minimum dietary diversity			
6–8 months	10 (45.5)	8 (42.1)	>0.999
9–24 months	50 (89.3)	30 (69.8)	0.020
Cumulative: 6–24 months	60 (76.9)	38 (61.3)	0.035
Minimum meal frequency			
6–8 months: ≥2 daily	13 (59.1)	9 (47.4)	0.538
9–24 months: ≥3 daily	11 (19.6)	9 (20.9)	>0.999
Cumulative: 6–24 months	24 (30.8)	18 (29.0)	0.486
Minimum acceptable diet			
6–8 months	7 (31.8)	5 (20.3)	0.744
9–24 months	11 (19.6)	8 (18.6)	>0.999
Cumulative: 6–24 months	18 (23.1)	13 (21.0)	0.464

a Chi‐square test, significance (*P* < 0.05).

### Anthropometric measurements of study children

Anthropometric data showed no significant difference among children participating in the two programs. Mean WAZ‐scores for children in the CBGP (−0.49 ± 1.41) and GMP (−0.65 ± 1.19) programs were low (*P* = 0.372). Although the prevalence of underweight was low among children in the CBGP program (8.1%) and medium in the GMP group (13.9%), the difference was insignificant (*P* = 0.154). Of 141 children on complementary feeding, 11.8% (CBGP: 7.7% vs. GMP: 16.1%, *P* = 0.119) were underweight.

### Predictors of child feeding according to minimum IYCF guidelines

In the full model, among caregivers who achieved optimum child feeding according to WHO minimum IYCF standards for their infants aged 6–24 months, the number of children less than 5 years owned by the caregiver was the only significant predictor among the known determining factors of optimum child feeding (UOR: 4.90; 95% CI: 138–17.34, *P* = 0.014). Caregivers who had not more than two children under the age of 5 years were almost five times more likely to optimally feed their 6–24 months old infants than caregivers who had above two children under 5 years. However, caregivers who enrolled their newborns within the postnatal period had 2.5 increased odds of optimally feeding their infants though it was insignificant (UOR: 2.51; 95% CI: 0.97–6.48, *P* = 0.057). The full univariate binary logistic regression model is presented in Table 6.

**Table 6:** Full univariate binary logistic regression model showing predictors of child feeding according to WHO IYCF indicators

Explanatory variables	OR_unadj_	95% CI	*P*‐value
Type of child growth promotion program			
CBGP	REF		
GMP	0.922	0.41–2.07	0.844
Age of caregiver			
>20 years	REF		
≤20 years	0.516	0.19–1.42	0.200
Educational level			
Senior high school & above	REF		
Junior high school & below	0.486	0.16–1.52	0.215
Marital status^a^			
Married	REF		
Single	0.657	0.18–2.44	0.530
Parity			
≤4 children	REF		
>4 children	0.751	0.15–3.67	0.724
No. of children <5 years owned by caregiver			
>2 children	REF		
≤2 children	4.896	1.38–17.34	0.014
Household size			
≤6 members	REF		
>6 members	0.712	0.19–2.66	0.613
Weekly food expenditure^b^			
>50 cedis	REF		
≤50 cedis	1.043	0.43–2.51	0.925
Socioeconomic status			
Low SES	REF		
High SES	0.627	0.27–1.44	0.269
Caregiver educated on IYCF at antenatal clinic^c^	1.650	0.34–7.96	0.533
Educated on IYCF at child welfare clinic^c^	1.661	0.35–7.93	0.524
Ever visited at home by program worker^c^	1.414	0.64–3.15	0.397
Caregiver's IYCF knowledge rating			
High knowledge	REF		
Fair knowledge	1.000	0.17–5.77	1.000
Poor knowledge	0.505	0.16–1.62	0.252
Age of child			
≥12 months	REF		
<12 months	0.970	0.39–2.42	0.949
Sex of child			
Female	REF		
Male	0.763	0.34–1.70	0.507
Child's birth weight			
≥2.5 kg	REF		
<2.5 kg	0.509	0.14–1.92	0.319
Underweight			
WAZ ≥2	REF		
WAZ <−2	1.173	0.35–3.93	0.796
When child was enrolled on the program			
Postnatal period	REF		
After postnatal period	2.509	0.97–6.48	0.057

a All caregivers who were living with their partners were regarded married.

b The exchange rate then was 1 US dollar = 1.8 Ghana cedi.

c The reference (OR = 1.00) is a “yes” response.

In the final model, presented in Table 7, again, number of children <5 years owned by the caregiver, educational level of the caregiver, and her IYCF knowledge rating were the explanatory variables in which significant associations were observed. The odds of caregivers with ≤2 children under 5 years optimally feeding their infants increased by additional 4.5 points in the final model (AOR: 0.405; 95% CI: 1.13–78.53, *P* = 0.038). Caregivers who were educated to only the junior the high school level had a significant 0.11 reducing odds of optimally feeding their infants on complementary feeding compared to those who were educated to at least the senior high school level (AOR: 0.112; 95% CI: 0.02–0.90, *P* = 0.040). Similarly, caregivers with fair knowledge on IYCF had a significant 0.14 reducing odds of optimally feeding their infants on complementary feeding compared to those with high IYCF knowledge (AOR: 0.140; 95% CI: 0.03–0.79, *P* = 0.026). Compared to the full model, increasing odds for optimal infant feeding were observed in two variables in the final model: caregiver's SES and when infant was enrolled on the child growth promotion program.

**Table 7:** Final multivariate binary logistic regression model showing interaction of the explanatory variables on achievement of optimum infant feeding

Explanatory variables	OR_adj_	95% CI	*P*‐value
Age of caregiver			
>20 years	REF		
≤20 years	0.283	0.03–2.46	0.253
Educational level			
Senior high school & above	REF		
Junior high school & below	0.112	0.02–0.90	0.040
No. of <5 year children owned by caregiver			
>2 children	REF		
≤2 children	9.405	1.13–78.53	0.038
Socioeconomic status			
Low SES	REF		
High SES	1.489	0.38–5.82	0.567
Home visit by a program worker	0.735	0.16–3.33	0.689
Caregiver's IYCF knowledge rating			
High knowledge	REF		
Fair knowledge	0.140	0.03–0.79	0.026
Poor knowledge	0.896	0.04–20.01	0.945
Child's birth weight			
≥2.5 kg	REF		
<2.5 kg	0.393	0.08–1.83	0.235
When child was enrolled on the program			
Postnatal period	REF		
After postnatal period	3.308	0.66–16.58	0.146

Hosmer–Lemeshow goodness of fit: Chi‐square = 10.820, *P* = 0.212.

## Discussion

Some differences were observed in the socio‐demographic variables of caregivers enrolled on the CBGP and GMP programs (age, educational level, marital status, parity, source of water, and type of fuel used for cooking). However, these did not translate into differences in the SES of the two groups. Caregivers participating on the CBGP programs were 4 years older than their GMP counterparts. Usually, persons who are relatively older tend to be in a marriage relationship, have higher parity, and attain higher level of education. The lower education of the GMP caregivers could probably be due to their relatively younger age. If age of caregivers in both groups were similar, it is possible their educational status would also be similar. However, the influence of educational status on caregivers child care giving and seeking behaviors cannot be over‐emphasized. After adjusting for caregiver–child pairs’ socio‐demographic factors, educational level was a significant determinant of optimum child feeding. This finding has been confirmed in other studies (Abuya et al. 2012; Bornstein et al. 2015). Mothers with at least secondary education have decreased likelihood of their infants being stunted (UOR: 1.28; CI: 1.096–1.498, *P* = 0.002) (Abuya et al. 2012).

Knowledge of study participants on appropriate breastfeeding and complementary feeding practices was generally good with minimal differences between the two groups. Although mean knowledge scores were similar for both groups, on the knowledge ranking scale, some interesting differences were observed; 87% of GMP caregivers were moderately informed on IYCF practices but this was 10 points less in the CBGP group. However, 17% of the CPGP caregivers were highly knowledgeable compared to only 5% in GMP group. Since both child growth promotion programs focus on improving the nutrition knowledge of caregivers, it is disappointing to report low IYCF knowledge among the caregivers as this influenced their IYCF practices. In a similar study in Honduras where the community nutrition program was assessed, mean knowledge scores of caregivers participating in the program was higher (6.02 out of 9 points) than that for the control communities (4.91; *P* = 0.001) (Van Roekel et al. 2002). However, it is worth noting that in this study, disparity between the mean knowledge scores and the knowledge rankings may be a result of the cut‐offs used.

Assessment of IYCF practices of caregivers revealed some differences. A significant proportion of CBGP caregivers translated their breastfeeding knowledge into practice having reported practicing early initiation of breastfeeding, exclusive breastfeeding and timely introduction of complementary diets. Effective caregiver counseling has been shown to increase breastfeeding rates (Bhutta et al. 2008; Arabi et al. 2012; Negash et al. 2014). Findings from this study also indicated that caregivers in the CBGP program fed their 6–24 months infants with varied food resources than their GMP counterparts but in both groups, the mean dietary diversity score was less than half of the 10 food groups investigated. Since the CBGP caregivers spend relatively more of their income on food per week, coupled with a higher number being married, employed and better educated, it appears these factors might have positively influenced the number of foods they fed their infants with. Consequently, a significant difference existed in the proportion of CBGP and GMP caregivers who met the IYCF guideline relating to minimum dietary diversity. But in both groups, just a quarter met the minimum meal frequency indicator. Therefore, over almost 80% caregivers did not achieve the minimum acceptable diet (optimum child feeding). This finding did not come as a surprise because in both groups, caregivers’ knowledge on minimum meal frequency was poor. They believed that young children should be fed three times, just like adults. Suboptimum complementary feeding has become a public health problem globally (Onyango et al. 2014). In Haiti, minimum dietary diversity, minimum meal frequency and minimum acceptable diet were achieved in 29.2%, 45.3%, and 17.1% of children aged 6–23 months, respectively (Heidkamp et al. 2013).

The child growth promoters were observed to render client‐centered counseling using counseling cards and often negotiated and agreed with the caregivers on the exact IYCF actions to take (data not reported in this paper). Literature attest that the success of child growth programs rely heavily on the crucial one‐to‐one counseling contact which caregivers and their family members receive from community workers (Hossain et al. 2005; Muyeti‐Stevens 2007; Imdad et al. 2011). Although this study did not prove that the type of growth promotion program that caregiver–child pairs participate in affect the IYCF practices, the caregivers IYCF knowledge has been shown to be a crucial predictor for optimum child feeding. Contrary to findings from Waswa et al. (2015), this study and others have shown that nutrition knowledge of caregivers positively predicts adequate dietary intakes of infants (Dewey and Adu‐Afarwuah 2008; Inayati et al. 2012). In Honduras, caregivers’ knowledge gained from counseling messages received in the community nutrition program was translated into practice evidenced by their higher child feeding scores (Van Roekel et al. 2002). In Senegal also, caregivers participating in a similar program exhibited significant gains in IYCF practices (Alderman et al. 2009). A year into its implementation, caregivers who practiced adequate complementary feeding increased from 35% to 72%.

Prevalence of underweight among children in the GMP program was similar to the 14% national prevalence recorded in the 2008 Ghana demographic and health survey (Ghana Statistical Service, 2008). However, there was 6 point decrease in the CBGP group. Breastfeeding practices of over half of the caregivers was appropriate but complementary feeding practices of over two‐thirds were suboptimum. Most studies have documented widespread growth faltering during the complementary feeding period where infant feeding practices become suboptimum (Heidkamp et al. 2013; Mishra et al. 2014). In both the unadjusted and adjusted binomial regression models, the number of children under 5 years owned by the caregiver was the significant predictor of optimum child feeding. Children under 5 years are vulnerable and require special care. Therefore, having more than two children <5 years would imply a reduction in the caregiving and care‐seeking attention rendered to each of them while the limited family food and other resources would have to be shared among these vulnerable ones. The result will be regular suboptimum feeding which will predispose such children to growth faltering. This is a significant finding with little literature from other studies to back it. However, maternal parity in general have been found to correlate with both child feeding practices and nutritional status (Abuya et al. 2012; Radwan 2013).

Poor nutrition knowledge of community health workers and volunteers could adversely affect program outcomes. This was the case with the community‐based growth monitoring program in South Africa (Faber et al. 2009). In developing countries, one of the current challenges to child nutrition is the considerable global and national efforts being devoted to breastfeeding promotion to the neglect of complementary feeding practices (Dewey and Adu‐Afarwuah 2008; Lartey 2008). From this study, however, it remains unclear whether the IYCF knowledge and practices of the caregivers was a result of the counseling received from the program facilitators. Also, whether the counseling yielded the intended impact of behavior change is an issue worth exploring. Another limitation of the study is the lack of information on nutrition knowledge of the community volunteers and health workers giving education to the caregivers thus making it difficult to assume a relationship between the nutrition knowledge and counseling provided.

**Key messages**

- Caregivers in the CBGP program were more knowledgeable on IYCF. Most caregivers in both programs practiced appropriate breastfeeding but could not meet the complementary feeding indicators.
- Underweight prevalence was lower in the CBGP program which suggests that the program has the potential to make significant contribution to child health in Ghana.
- Caregivers with fewer children <5 years, high IYCF knowledge and higher education had increased odds for optimum infant feeding.
- Counseling of caregivers and their families on adequate complementary feeding and birth spacing should intensify and should be integrated into all maternal and child health interventions.
- Both programs should be strengthened to enhance their outcomes

### Lesson learned and recommendations

Findings from this study provide a firm basis for strengthening and expansion of the CBGP program to augment the traditional GMP program in Ghana as it addresses some short‐falls in the traditional GMP program such as high prevalence of underweight and poor IYCF knowledge. Addressing some operational challenges in the CBGP and GMP programs would help make both programs more effective. Capacity of child growth promoters (community volunteers), community health nurses and other categories of health workers who provide child welfare services should be built through regular training on evidenced‐based contemporary IYCF recommendations. They should also be equipped with modern behavior change communication skills to appropriately deliver these messages to the target audience. In filling the knowledge deficit gap, special attention should be given to dietary diversification, food consistency, quantity of meals and feeding frequency. Since infants are particularly vulnerable during the transition period, counseling on child feeding should provide core information on timeliness, adequacy, safety and proper preparation of complementary diets. Emphasis should be placed on involvement of caregivers to understand their challenges to meeting complementary feeding recommendations and where possible, solutions offered to suit their child(ren) and family needs. Finally, since the CBGP program is not meant to replace the traditional GMP program, the Ghana Health Service should take pragmatic steps to strengthen the institutional systems within the GMP program to make it relevant to the needs of caregivers, families and communities.

## Conflict of Interest

None declared.
